# Management Strategies for Hyperglycemia Associated with the α-Selective PI3K Inhibitor Alpelisib for the Treatment of Breast Cancer

**DOI:** 10.3390/cancers14071598

**Published:** 2022-03-22

**Authors:** Tsvetalina Tankova, Elżbieta Senkus, Maria Beloyartseva, Simona Borštnar, Doina Catrinoiu, Mona Frolova, Alinta Hegmane, Andrej Janež, Mladen Krnić, Zoltan Lengyel, Yiola Marcou, Laura Mazilu, Bela Mrinakova, Ruth Percik, Katarina Petrakova, Gábor Rubovszky, Margarita Tokar, Eduard Vrdoljak

**Affiliations:** 1 Department of Endocrinology, Medical University of Sofia, 2, Zdrave Str., 1431 Sofia, Bulgaria; 2 Department of Oncology & Radiotherapy, Medical University of Gdańsk, Smoluchowskiego 17, 80-214 Gdańsk, Poland; elsenkus@gumed.edu.pl; 3 Institution N.N. Blokhin National Medical Research Center of Oncology of the Ministry of Health of the Russian Federation, 23 Kashirskoye Avenue, 115478 Moscow, Russia; m.beloyartseva@ronc.ru (M.B.); drfrolova@yandex.ru (M.F.); 4 Division of Medical Oncology, Institute of Oncology Ljubljana, Zaloska 2, 1000 Ljubljana, Slovenia; sborstnar@onko-i.si; 5 Department of Diabetology, Clinical Emergency Hospital of Constanta, Romania, Tomis Bvd. No. 145, 900591 Constanta, Romania; dcatrinoiu@gmail.com (D.C.); laura.mazilu@univ-ovidius.ro (L.M.); 6 Faculty of Medicine, “Ovidius” University of Constanta, University Alley No. 1, 900470 Constanta, Romania; 7 Out-Patient Department of Medical Oncology, Riga East University Hospital, Oncology Center of Latvia, 4, Hipokrata Str., LV1079 Riga, Latvia; alinta.hegmane@aslimnica.lv; 8 Department of Endocrinology, Diabetes and Metabolic Disease, University Medical Center, Zaloska 7, 1000 Ljubljana, Slovenia; andrej.janez@kclj.si; 9 Department of Endocrinology, Clinical Hospital Center Split, School of Medicine, University of Split, Šoltanska 1, 21000 Split, Croatia; mladen.krnic@mefst.hr; 10 Szent János Hospital, Diós árok 1-3, 1125 Budapest, Hungary; dr.lengyel.zoltan@sztmargit.hu; 11 Medical Oncology Department, The Bank of Cyprus Oncology Centre, 32 Acropoleos Avenue, Strovolos, Nicosia 2006, Cyprus; yiola.marcou@bococ.org.cy; 12 1st Department of Oncology, Comenius University, Faculty of Medicine, Bratislava, Heydukova 10, 812 50 Bratislava, Slovakia; bela.mrinakova@ousa.sk; 13 Slovak Republic Department of Medical Oncology, St. Elisabeth Cancer Institute, Heydukova 10, 812 50 Bratislava, Slovakia; 14 Division of Endocrinology, Diabetes and Metabolism, Sheba Medical Center, Tel-Hashomer, Ramat Gan 52621, Israel; ruth.percik@sheba.health.gov.il; 15 Sackler Faculty of Medicine, Tel Aviv University, P.O. Box 39040, Ramat Aviv, Tel Aviv 69978, Israel; 16 Masaryk Memorial Cancer Institute, Žlutý kopec 543/7, Brno-Střed-Staré, 602 00 Brno, Czech Republic; petrakova@mou.cz; 17 National Institute of Oncology, Rath Gy. Str. 7-9, 1122 Budapest, Hungary; rubovszky.gabor@oncol.hu; 18 The Legacy Heritage Oncology Center and Dr. Larry Norton Institute, SorokaMedical Center, Yitzhack I. Rager Blvd 151, Be’er Sheva, Israel; ritato@clalit.org.il; 19 Department of Oncology, Clinical Hospital Center Split, School of Medicine, University of Split, Spinčićeva 1, 21000 Split, Croatia; vrdoljak@kbsplit.hr

**Keywords:** adverse effect, alpelisib, hyperglycemia, *PIK3CA*-mutated metastatic breast cancer

## Abstract

**Simple Summary:**

Alpelisib is a drug used to treat breast cancer that has certain characteristics (hormone receptor-positive (HR+), human epidermal growth receptor 2-negative (HER2–), *PIK3CA*-mutated) and that has or has not spread to other organs. It is used after the cancer has progressed despite being treated with hormonal therapies. One of the most common side effects of alpelisib is an increase in blood glucose level (hyperglycemia). This can sometimes require reductions in the dose of alpelisib, or interruption or discontinuation of treatment. Early detection and initiation of treatment for hyperglycemia can help in controlling blood glucose levels and ensuring the best use and effects of alpelisib. Treatment can include lifestyle modifications (a reduced-carbohydrate diet) and administration of drugs used to treat diabetes. This report provides information on how to manage hyperglycemia caused by alpelisib, based on the experience of 14 cancer specialists and seven endocrinologists in managing this side effect.

**Abstract:**

Alpelisib is an α-selective phosphatidylinositol 3-kinase inhibitor used for treating hormone receptor-positive (HR+), human epidermal growth receptor 2-negative (HER2–), *PIK3CA*-mutated locally advanced or metastatic breast cancer following disease progression on or after endocrine therapy. Hyperglycemia is an on-target effect of alpelisib affecting approximately 60% of treated patients, and sometimes necessitating dose reductions, treatment interruptions, or discontinuation of alpelisib. Early detection of hyperglycemia and timely intervention have a key role in achieving optimal glycemic control and maintaining alpelisib dose intensity to optimize the benefit of this drug. A glycemic support program implemented by an endocrinology–oncology collaborative team may be very useful in this regard. Lifestyle modifications, mainly comprising a reduced-carbohydrate diet, and a designated stepwise, personalized antihyperglycemic regimen, based on metformin, sodium–glucose co-transporter 2 inhibitors, and pioglitazone, are the main tools required to address the insulin-resistant hyperglycemia induced by alpelisib. In this report, based on the consensus of 14 oncologists and seven endocrinologists, we provide guidance for hyperglycemia management strategies before, during, and after alpelisib therapy for HR+, HER2–, *PIK3CA*-mutated breast cancer, with a focus on a proactive, multidisciplinary approach.

## 1. Introduction

Endocrine therapy, with or without the use of a cyclin-dependent kinase 4 and 6 (CDK4/6) inhibitor, is the current standard of care for patients with advanced hormone receptor-positive (HR+), human epidermal growth factor receptor 2-negative (HER2–) breast cancer [1,2]. However, acquired resistance to endocrine-based therapy remains a major challenge [3,4,5]. Activating mutations in the PIK3 catalytic subunit alpha (*PIK3CA*) gene, which encodes the p110α-isoform of phosphatidylinositol 3-kinase (PI3K), occur in approximately 40% of patients with advanced HR+, HER2– breast cancer [6,7,8,9], and are associated with resistance to endocrine-based therapy and poor prognosis [6,10,11,12].

Alpelisib is a small-molecule α-selective PI3K inhibitor that can help overcome resistance to endocrine-based therapy [9,13,14,15,16]. Alpelisib is the only PI3K inhibitor approved for the treatment of HR+, HER2–, *PIK3CA*-mutated advanced or metastatic breast cancer that has progressed on or after endocrine therapy. Regulatory approval of alpelisib, and its inclusion in breast cancer treatment guidelines [1,2], was based on the phase 3 SOLAR-1 study of alpelisib (300 mg/day) plus fulvestrant versus placebo plus fulvestrant in patients with HR+, HER2– breast cancer that had progressed on or after aromatase inhibitor (AI) therapy [9]. In patients with *PIK3CA*-mutated disease, there was a clinically relevant and statistically significant benefit on progression-free survival (PFS), the primary endpoint, with alpelisib plus fulvestrant (median PFS 11 vs. 5.7 months; hazard ratio (HR), 0.65; 95% confidence interval (CI), 0.5–0.85; *p* < 0.001) [9] and a 7.9-month numeric improvement in median overall survival (HR, 0.86; 95% CI, 0.64–1.15; *p* = 0.15) [13]. In an unplanned, exploratory analysis, the PFS benefit of alpelisib versus placebo was evident irrespective of dose intensity, but a higher median dose intensity (≥248 mg/day) resulted in a longer median PFS compared with lower dose intensity (<248 mg/day), 12.5 vs. 9.6 months, respectively, which suggests an important role for dose intensity in achieving maximum treatment benefit [17].

Results from the more recent phase 2 BYLieve study indicate that alpelisib in combination with endocrine therapy (fulvestrant or letrozole) is also effective in patients with HR+, HER2–, *PIK3CA*-mutated breast cancer in the post-CDK4/6 inhibitor setting [16,18]. The proportion of patients alive without disease progression at 6 months (primary endpoint) was 50.4% (95% CI, 41.2–59.6) in patients treated with alpelisib plus fulvestrant who had previously progressed on a CDK4/6 inhibitor plus an AI [16], and 46.1% (95% CI, 36.8–56.6) in patients treated with alpelisib plus letrozole who had previously progressed on a CDK4/6 inhibitor plus fulvestrant; this met the prespecified threshold of clinically meaningful efficacy specified as the lower boundary of the 95% CI of >30% [18].

In contrast to pan-PI3K inhibitors, development of which was limited by toxicity [19,20,21], alpelisib can be administered safely in patients with HR+, HER2–, *PIK3CA*-mutated breast cancer, although adverse events (AEs) associated with PI3K inhibition are common [17,22]. Hyperglycemia is a particularly challenging on-target class effect of PI3K inhibitors [17,23], and was the most common all-grade AE reported in patients treated with alpelisib in clinical trials, with a random-effect absolute risk of 59% (95% CI, 0.51–0.66) [22]. With the exception of hyperglycemia, treatment-emergent AEs occurring in patients treated with alpelisib in clinical trials were generally low-grade [9,22]. The absolute risk of grade 3/4 hyperglycemia was 28% (95% CI, 0.21–0.37), whereas all other grade 3/4 AEs occurred in ≤10% of patients [22].

**Figure 1:**
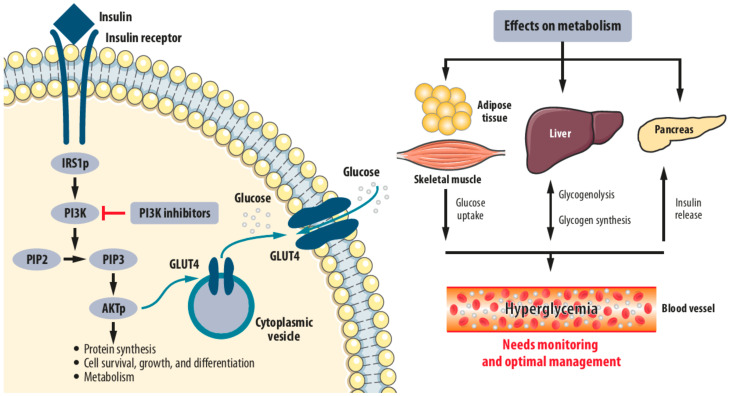
PI3K inhibitors prevent the conversion of PIP2 to PIP3 and the activation of AKT through phosphorylation by PIP3. Blockade of the metabolic effects of AKT leads to (1) transient insulin resistance due to lack of translocation of the glucose transporter GLU4 from cytoplasmic vesicles to the cell membrane, and therefore lack of passage of glucose molecules into the liver, fat, and muscle cells, as well as (2) increased glycogenolysis in the liver. In turn, both (1) and (2) lead to hyperglycemia, as well as increased insulin release by the pancreas with ensuing hyperinsulinemia. AKT, protein kinase B; GLU4, glucose transporter 4; IRS1p, insulin receptor substrate 1, phosphorylated; PI3K, phosphatidylinositol 3-kinase PIP2, phosphatidylinositol 4,5-bisphosphate; PIP3, phosphatidylinositol 3,4,5-trisphosphate.

Hyperglycemia occurs early in the course of treatment (median time of onset about 2 weeks from initiation of alpelisib therapy [17]) because inhibition of PI3K-α blocks the metabolic actions of insulin, preventing glucose uptake in skeletal muscle and adipose tissue and promoting hepatic glycogenolysis, which results in increased blood glucose levels and a compensatory release of insulin (Figure 1) [6,24,25,26,27].

The hyperinsulinemia that occurs in patients with alpelisib-induced hyperglycemia may provide breast cancer cells with a survival mechanism and reduce the efficacy of alpelisib as demonstrated in preclinical studies [6,23,25,28,29]. Furthermore, if not successfully managed, hyperglycemia can necessitate alpelisib dose reductions, treatment interruptions, or treatment discontinuation [9,17,23,30,31]. Optimal hyperglycemia management is therefore required to maintain alpelisib dose intensity and maximize treatment benefit [17]. Fortunately, alpelisib-induced hyperglycemia is predictable, readily identifiable, and generally manageable [17,23,24], but pragmatic guidelines could help improve the management of this challenge [24].

In this report, we provide guidance for hyperglycemia management and monitoring strategies aimed at minimizing alpelisib dose disruption in patients with HR+, HER2−, *PIK3CA*-mutated advanced breast cancer.

## 2. Methods and Data Sources

The guidance provided in this report is the consensus opinion of a group of 14 oncologists and seven endocrinologists, who are all experts in oncology and endocrinology/diabetology from leading academic and non-academic institutions in 12 countries in Central/Eastern Europe and Israel. The content is based on a review of the published literature, recommendations on the management and monitoring of alpelisib-induced hyperglycemia from the alpelisib prescribing information, and shared inter-specialty experience of the aforementioned oncologists and endocrinologists in the management of alpelisib-induced hyperglycemia. Drafts of the guidance were reviewed and modified by this expert group the until consensus was reached.

## 3. Preventive Management Strategies before Starting Alpelisib

Pretreatment strategies for the prevention of hyperglycemia in breast cancer patients scheduled to receive alpelisib are listed in Table 1. Before starting treatment with alpelisib, it is important that all patients receive a thorough hyperglycemia risk assessment, and that high-risk patients are referred to an endocrinologist/diabetologist for ongoing care. All patients require lifestyle management advice on how to minimize the risk of alpelisib-induced hyperglycemia. 

### 3.1. Risk Assessment

Patients should be screened for the following hyperglycemia risk factors before starting treatment with alpelisib: prediabetes, diabetes, body mass index (BMI), and age. Diabetes is diagnosed based on fasting plasma glucose (FPG) and glycated hemoglobin (HbA1c) levels according to American Diabetes Association (ADA) criteria [32]. We consider a history of gestational diabetes as another risk factor, because it implies a baseline propensity to insulin resistance.

**Figure 2:**
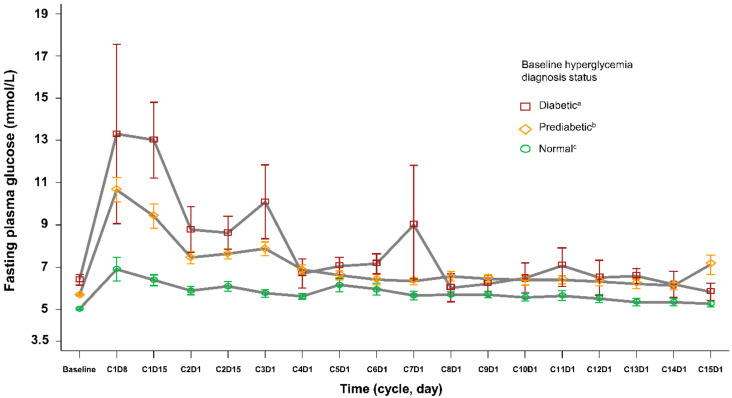
Changes in mean FPG over time according to glycemic status in patients with hormone receptor-positive breast cancer receiving alpelisib plus fulvestrant in the SOLAR-1 trial. Adapted with permission from Ref. [17], Copyright 2020 Elsevier. ^a^ FPG > 7.0 mmol/L (>126 mg/dL) and/or HbA1c > 6.5%; ^b^ FPG 5.6 to <7.0 mmol/L (100 to <126 mg/dL) and/or HbA1c 5.7 to <6.5%; ^c^ FPG < 5.6 mmol/L (<100 mg/dL) and HbA1c < 5.7%. FPG: fasting plasma glucose; HbA1c: glycated hemoglobin.

Our risk assessment recommendations are based on the findings of the SOLAR-1 study, which included patients with a history of well-controlled type 2 diabetes [9,17]. Despite initial exclusion of patients with uncontrolled type 2 diabetes, a protocol amendment resulted in a small proportion of patients in the alpelisib treatment group (4%) having a baseline glycemic status indicative of type 2 diabetes (FPG ≥ 7.0 mmol/L (≥126 mg/dL) and/or HbA1c ≥ 6.5%), and 56% of patients being considered prediabetic (FPG 5.6 to <7.0 mmol/L (100 mg/dL to <126 mg/dL) and HbA1c 5.7 to <6.5%). Compared with patients with normal baseline glycemic status (FPG < 5.6 mmol/L (<100 mg/dL) and HbA1c < 5.7%; *n* = 113), increases in FPG occurring during alpelisib therapy were more pronounced in patients with diabetic or prediabetic glycemic status at baseline (Figure 2), and there was a higher incidence of any-grade hyperglycemia during alpelisib treatment in prediabetic patients (74%) than in those with normal glycemic status (52%) [17]. In patients with normal FPG at baseline, FPG tended to increase only in the first cycle of alpelisib treatment, whereas in patients with prediabetes or diabetes, FPG increases occurred during subsequent cycles as well (Figure 2). Regardless of baseline hyperglycemia status, mean FPG levels generally normalized over time, which was most likely attributable to antidiabetic medications (e.g., metformin and/or insulin), which were used to manage blood glucose levels in 163 of 187 patients (87%) with hyperglycemia, and early discontinuation of alpelisib due to hyperglycemia, which occurred in 18 of 284 patients (6%) in the alpelisib treatment group [9,17].

In addition to diabetic or prediabetic glycemic status at baseline, BMI and age also appeared to be risk factors for the development of hyperglycemia in patients receiving alpelisib in the SOLAR-1 study [17]. Hyperglycemia occurred during alpelisib therapy in 57% of patients with a normal BMI versus 68–74% of patients who were overweight or obese, and the incidence of grade 3/4 hyperglycemia was higher in patients aged ≥ 75 years than in younger patients (55% vs. 36%) [17].

### 3.2. Preventive Strategies

Given that hyperglycemia occurred in >50% of patients with normal glycemic status and normal BMI who received alpelisib in the SOLAR-1 study [17], all patients should be instructed to adjust their diet in line with the ADA guidelines to minimize the risk of developing hyperglycemia during alpelisib treatment [33]. A high-fiber diet rich in vegetables, legumes, whole grains, and dairy (milk and yogurt) products is recommended, with carbohydrate intake limited to ~30–40% of daily calorie intake. Simple carbohydrates (sugary drinks, juices, pastry, sweets, cookies, candy, fruits) should be avoided and replaced with complex carbohydrates (whole-grain bread, cereals, grains, potatoes, pasta, beans, porridge, dairy, gruel). Generally speaking, patients should be advised to eat a balanced diet with as low a carbohydrate intake as the patient can tolerate, since not all patients can tolerate the same (low) amount of carbohydrate and not all patients need the same daily intake of carbohydrate. 

There are some preclinical data to suggest that ketogenic diets may enhance the efficacy of PI3K inhibitors and mTOR inhibitors by depleting hepatic glycogen stores and reducing blood glucose and insulin levels [28,34,35,36]. However, such diets are not usually recommended as they may not be well tolerated or sustainable and can lead to positive urine ketones, which may be misinterpreted as alpelisib-induced ketoacidosis. A preclinical study investigating PI3K inhibitors in combination with a ketogenic (low-carbohydrate) diet found that this led to drastic deterioration in the general health condition of the experimental animals [28]. The ketogenic diet resulted in the best therapeutic impact on tumor xenotransplants, but the worst effect on animal well-being [28]. Therefore, it should not be recommended for people before more data on the safety of such an approach are available. On the other hand, ketones in the blood are a strict indication for insulin therapy initiation. Therefore, a ketogenic diet may misleadingly suggest ketoacidosis, either induced by alpelisib itself or by an SGLT2 inhibitor if this treatment has been used for hyperglycemia. Finally, a separate preclinical study in a murine model of animals without cancer found that a short-term ketogenic diet induces more severe hepatic insulin resistance than an obesogenic high-fat diet [37]. A clinical trial is currently underway on the effects of a very-low-carbohydrate diet on rates of hyperglycemia in subjects treated with alpelisib (NCT05090358). Until these results are available and for the reasons described above, we do not recommend very-low-carbohydrate diets, but rather moderate carbohydrate restriction.

Meal planning needs to be individualized based on body weight and other variables, such as cardiovascular risk and level of glycemia. Wherever possible, personal preferences and needs should be accommodated, as long as they correspond with a low-sugar diet (i.e., no sugar-sweetened drinks, fruit juices, pastries, jams, desserts) [6]. Physical activity should be encouraged but tailored to the patient’s performance status and capabilities.

We recommend that high-risk patients (i.e., those with prediabetes or diabetes, especially if BMI is ≥30 kg/m^2^ or in those aged ≥ 75 years) are managed by a multidisciplinary team that includes, whenever possible, an endocrinologist or diabetologist to oversee appropriate antihyperglycemic treatment and ongoing management [24]. When patients are at high risk of developing hyperglycemia but without possibility of timely referral to an endocrinologist, the oncologist and patient should discuss the risks and benefits of alpelisib therapy and together make an informed decision. In high-risk patients, alpelisib should be administered with caution, and in accordance with available management recommendations, including these in the current manuscript, always considering the patient’s safety as the absolute priority. As alpelisib may lead to life-threatening hyperglycemia within 2–3 days in patients with diabetes, oncologists should prescribe it with extreme care in these patients and preferably in consultation with an endocrinologist or diabetologist.

The antihyperglycemic medication metformin, which increases insulin sensitivity and reduces hyperglycemia and insulin levels, is commonly used in patients with diabetes, and is safe and widely accepted for use in prediabetes [38,39,40]. Metformin has been used to prevent, or at least delay, the onset of diabetes in patients considered to be at high risk for the disease [41]. The development of insulin resistance, in which the action of insulin on glucose metabolism is blunted, occurs early in the pathogenesis of dysglycemia. The (2015) Position Statement from the American Diabetes Association recommends that metformin has the strongest evidence base of pharmacological agents for diabetes prevention [42]. Moreover, metformin may have some antitumor activity [43].

Given the early onset and frequency of hyperglycemia associated with alpelisib, a pre-emptive approach to management is recommended. Although there is currently only limited evidence supporting this practice [44], metformin along with lifestyle changes can be considered for the prevention of alpelisib-induced hyperglycemia in prediabetic patients or high-risk patients with normal baseline glycemic status. According to a recent review by Anders et al., many oncologists initiate 500 mg of metformin extended release (XR) 7 days before starting alpelisib in high-risk patients [24]. If metformin is used to prevent alpelisib-induced hyperglycemia in high-risk patients, gastrointestinal (GI) AEs associated with this drug need to be considered. The XR formulation of metformin has been proven to have fewer GI side effects than the immediate-release formulation and, thus, the use of the XR formulation may help to minimize overlapping toxicities of diarrhea and nausea associated with both alpelisib and metformin [24]. Studies are currently underway to investigate metformin for the prevention of alpelisib-induced hyperglycemia in breast cancer patients with or without prediabetes (NCT04300790 and NCT04899349). Given its insulin-lowering effects, the use of metformin to prevent and/or treat alpelisib-induced hyperglycemia may also enhance the anticancer effects of alpelisib [29].

### 3.3. Education

In general, before starting alpelisib, all patients and their treating physicians need to understand that hyperglycemia is a common AE during alpelisib therapy, and that possible prevention by dietary and physical activity approaches, early detection, and prompt treatment of hyperglycemia are key to successful ongoing alpelisib therapy [17,23]. Treating physicians need to make sure that patients are aware of the risk of developing hyperglycemia while receiving alpelisib. Patients must be advised of the signs and symptoms of hyperglycemia, such as excessive thirst, dry mouth, and high frequency or volume of urination [31,39], and told to contact their treatment team immediately in the event that any of these symptoms develop.

## 4. Management Strategies during Alpelisib Treatment

**Figure 3:**
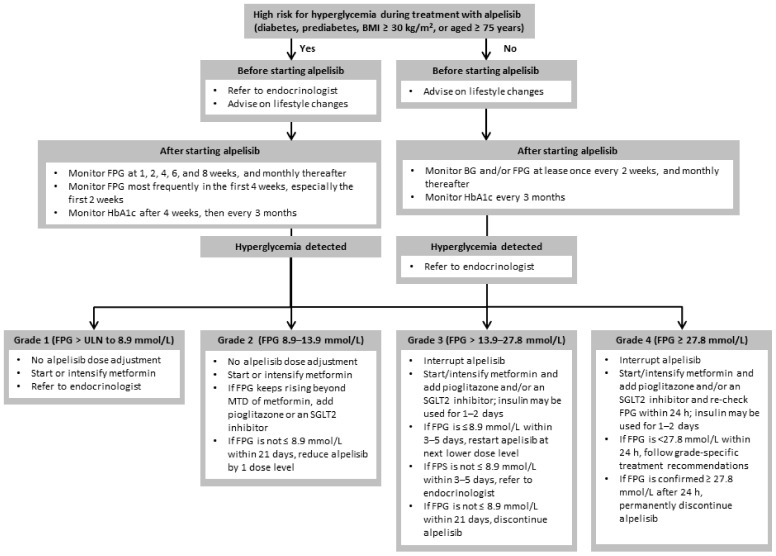
Algorithm for the monitoring and management of alpelisib-induced hyperglycemia. If positive for ketones, discontinue oral agents, and start insulin and intravenous hydration. BG: blood glucose; BMI: body mass index; FPG: fasting plasma glucose; HbA1c: glycated hemoglobin; hrs: hours; IV: intravenous; MTD: maximum tolerated dose; SGLT2: sodium–glucose co-transporter 2.

Alongside prevention, early detection and prompt treatment of hyperglycemia are crucial to maintaining alpelisib dose intensity and maximizing clinical benefit. If hyperglycemia is managed properly, most patients should be able to continue alpelisib without dose adjustment or interruption. Unlike grade 3/4 hyperglycemia, which requires alpelisib dose interruption and may mandate permanent discontinuation of treatment, grade 1/2 hyperglycemia is usually asymptomatic and manageable without alpelisib dose adjustment (Figure 3) [17,23,31]. Early identification of hyperglycemia and pharmacologic intervention to limit the onset of grade 3/4 hyperglycemia are, therefore, essential to facilitate alpelisib treatment continuation [17].

After amendment of the SOLAR-1 study protocol to improve hyperglycemia monitoring and management, the incidence of grade 3/4 hyperglycemia in the alpelisib treatment group was reduced from 40.3% to 32.9%, and discontinuation of alpelisib because of hyperglycemia became less frequent (9.0% vs. 3.6%) [17]. Furthermore, the overall rate of discontinuations due to hyperglycemia in patients treated with alpelisib in the more recent BYLieve study (1.6%) [16] was lower than in SOLAR-1 (6.3%) [9], indicating that experience in monitoring and managing AEs has improved as a result of better education and implementation of improved management strategies [13]. The hyperglycemia management guidelines in the amended SOLAR-1 protocol, which were based on recommendations by an advisory board of experts in managing hyperglycemia, included the provision of instruction on lifestyle changes at screening, frequent monitoring to identify hyperglycemia during treatment, and active management of hyperglycemia with metformin and other antihyperglycemic agents [17]. 

### 4.1. Hyperglycemia Monitoring

Given the high risk of treatment-emergent hyperglycemia, which occurred in 64% of patients receiving alpelisib plus fulvestrant in SOLAR-1 [9], FPG and HbA1c should be closely and routinely monitored during alpelisib therapy (Figure 3) [17,22,31]. Hyperglycemia can appear early after starting treatment (median time to onset of grade 3/4 hyperglycemia was 15 days in SOLAR-1; range, 5–395 days), so FPG monitoring should begin during the first week of treatment [17,31]. If baseline FPG is normal (<5.6 mmol/L (<100 mg/dL)) and patients are not in a high-risk category, monitoring can be performed by an oncologist. Patients with diabetes may require intensified antihyperglycemic treatment if blood glucose levels remain elevated, and preferably should be closely monitored by an endocrinologist or diabetologist, as should other high-risk patients.

The following glucose monitoring schedule is recommended for use in everyday practice, with more frequent monitoring considered in high-risk patients. After initiation of alpelisib, blood glucose should be monitored at least once every week in the first 2 weeks and at least once every 4 weeks thereafter by assessment of capillary blood glucose in fingerprick samples or by assessing FPG in a peripheral blood sample; HbA1c should be monitored after 4 weeks and every 3 months thereafter [31]. FPG is the preferred parameter for monitoring hyperglycemia related to alpelisib; however, if the patient returns an abnormal postprandial plasma glucose (PPG) result, they should undergo a complete glycemic assessment (FPG, PPG, and HbA1c). If available, self-monitoring and continuous glucose monitoring devices should be prescribed or recommended to patients. If hyperglycemia develops after starting alpelisib, blood glucose and/or FPG should be monitored regularly (at least twice a week until blood glucose or FPG returns to normal, then continued at least once a week for 8 weeks, and once every 2 weeks thereafter) in parallel with endocrinologist- or diabetologist-guided antihyperglycemic therapy and lifestyle changes [31].

### 4.2. Oral Antihyperglycemic Treatment

Metformin is widely accepted as a standard treatment for alpelisib-induced hyperglycemia [13,17,45]. As hyperinsulinemia usually accompanies even mild hyperglycemia, and may reduce the efficacy of alpelisib [25,26,27,28], alpelisib-induced elevations in FPG to >7 mmol/L (>126 mg/dL) should prompt initiation of metformin 500 mg/day titrated to a maximum dose of 2000 mg/day [17,23,24]. Other oral antihyperglycemic drugs that can be used in combination with metformin include the insulin sensitizer pioglitazone and sodium–glucose co-transporter 2 (SGLT2) inhibitors (Table 2) [17,23,24].

SGLT2 inhibitors inhibit the glucose transporters responsible for reabsorption of glucose in the kidney [46]. The resulting glycosuria lowers the plasma glucose level and decreases hyperinsulinemia [46]. Based on this mechanism of action, SGLT2 inhibitors represent a potential option for the treatment of alpelisib-induced hyperglycemia [17,31,47]. Indeed, preclinical data suggest that SGLT2 inhibitor therapy may reduce alpelisib-induced hyperglycemia to a greater extent than metformin does [28]. However, in contrast to metformin, SGLT2 inhibitors should be prescribed with caution because of some safety concerns [46].

In general, insulin sensitizers (e.g., metformin and pioglitazone) are preferable to insulin secretagogues (e.g., sulfonylureas, meglitinides) due to the deleterious effect of insulin on the PI3K blockade, as demonstrated in animal models [6,17]. Although metformin is considered a very safe antihyperglycemic agent that does not directly cause hypoglycemia [48], caution is advised in relation to the risk of diarrhea with metformin and alpelisib. Referral to an endocrinologist or diabetologist for antihyperglycemic treatment is strongly advocated, especially for patients with grade 3/4 hyperglycemia [23,24].

In SOLAR-1, metformin was used either alone or in combination with other antihyperglycemic agents in 87.1% of patients with hyperglycemia [17]. After peaking within the first 2 weeks of study treatment, mean FPG decreased toward baseline following initiation of antihyperglycemic therapy (Figure 2) [17]. Overall, glycemic control was generally rapid after the start of antihyperglycemic therapy, with a median of 6 days for improvement of hyperglycemia by ≥1 grade (range 4–7 days) [17].

Although diarrhea may be an AE of both alpelisib and metformin [17,49], the incidence and severity of diarrhea was comparable in patients who did and did not receive concomitant metformin in SOLAR-1 [17]. To avoid GI AEs, the metformin dose should be titrated gradually [45,49]. Maintenance of alpelisib therapy, rather than metformin, is preferred in cases of diarrhea. Metformin dose reduction or discontinuation with gradual re-challenge or a switch to the XR formulation could lead to improved tolerability [49], but in severe cases of diarrhea, a switch to pioglitazone or an SGLT2 inhibitor may be required. Dietary measures and the prophylactic use of probiotics should also be considered [50]. Loperamide may be considered for patients with ≥4 stools daily.

As is recommended in patients with diabetes [39], antihyperglycemic medications should be added to metformin in a stepwise manner to achieve glycemic control in patients with alpelisib-induced hyperglycemia (Table 2). In SOLAR-1, ≥3 antihyperglycemic medications were required in 28.8% of patients with alpelisib-induced hyperglycemia [17]. If the metformin dose is maximized and FPG does not improve to the desired level (<8.9 mmol/L (<160.2 mg/dL)), addition of the insulin-sensitizing agent pioglitazone, which has a complementary mechanism of action to metformin [51], can be considered [17,23,24]. Pioglitazone cannot be used as a first-line drug because of delayed onset of action (6–8 weeks) [52], but it can be introduced in conjunction with metformin for grade 3/4 hyperglycemia. Based on their mechanism of action, SGLT2 inhibitors have been proposed as the second-line, and in cases of intolerance to metformin potentially first-line, treatment of choice. Although more data are needed to support the use of SGLT2 inhibitors as a routine first- or second-line treatment strategy [17,31,47], initial experience is encouraging. Data are available from six patients enrolled in the SOLAR-1 trial who received an SGLT2 inhibitor (empagliflozin, ipragliflozin, or dapagliflozin) in addition to metformin; this resulted in a stabilization of blood glucose levels and allowed the patients to continue to receive alpelisib [53]. Going forward, it will be important to conduct clinical trials to investigate the efficacy of SGLT2 inhibitors in the treatment of alpelisib-induced hyperglycemia, and their effect on the duration of, exposure to, and efficacy of alpelisib [54].

Sulfonylureas should be avoided as primary treatment for alpelisib-induced hyperglycemia because they act as insulin secretagogues and increase insulin levels, which may blunt the primary antitumor effect of alpelisib [28]. Insulin therapy, and/or subsequent sulfonylureas, are acceptable as rescue therapy, but only after treatment with more suitable hypoglycemic agents has proven insufficient. 

### 4.3. Insulin

After interruption of alpelisib, short-term insulin (1–2 days) can be used as rescue medication for grade 3/4 hyperglycemia (FPG > 13.9–27.8 mmol/L (>250–500 mg/dL)) not controlled by oral antihyperglycemic medications alone (Figure 3) [17,23,30]. Given the short half-life of alpelisib (8–9 h at steady state with 300 mg/day), blood glucose levels tend to normalize within 24–72 h after treatment is interrupted [17,24,31,55], so insulin may not be necessary and ongoing insulin (>1–2 days) is generally not required [23]. Alpelisib can be resumed at a lower dose if hyperglycemia resolves to grade ≤ 1 (FPG ≤ 8.9 mmol/L (≤160 mg/dL)) within 3–5 days of appropriate treatment with oral antihyperglycemic agents (±insulin), but referral to an endocrinologist or diabetologist is recommended if grade ≥ 2 hyperglycemia persists [30]. If grade 4 hyperglycemia persists beyond 24 h of interrupting alpelisib, or grade 3 hyperglycemia does not improve to grade ≤ 1 within 21 days of starting aggressive antihyperglycemic treatment, permanent discontinuation of alpelisib is recommended (Figure 3) [17,24,30,31,55].

Whenever possible, use of insulin should be avoided in patients with alpelisib-induced hyperglycemia because of the risk of hypoglycemia as the effect of the drug subsides [23,31,45,56]. However, insulin is required for ketoacidosis (Table 2), which can occur in the alpelisib treatment setting, albeit rarely, and usually in association with undetected and untreated hyperglycemia, further highlighting the importance of regular blood glucose monitoring during alpelisib treatment [23,31,45,56]. Patients with grade 3/4 hyperglycemia should be routinely evaluated for ketones, and if positive, oral antihyperglycemic therapy should be discontinued and aggressive treatment with insulin and intravenous hydration is required in the inpatient setting [24,56].

## 5. Management Strategies after Alpelisib Treatment

Hyperglycemia is a reversible AE of alpelisib [17], with blood glucose levels expected to normalize within 1 week of treatment withdrawal [17,24,31,55]. Among patients in the SOLAR-1 study who discontinued alpelisib because of elevated FPG, almost all (96%) had FPG levels that returned to baseline after alpelisib was discontinued [31].

The management approach to hyperglycemia upon discontinuation of alpelisib depends on the glycemic status of patients before starting alpelisib. In patients with diabetes or prediabetes, ongoing blood glucose monitoring will indicate when to change or discontinue antihyperglycemic treatment. In other patients with alpelisib-induced hyperglycemia, antihyperglycemic treatment should be discontinued once FPG levels have normalized. There is a risk of hypoglycemia if alpelisib is stopped suddenly during oral antihyperglycemic therapy. Bearing in mind that FPG levels may normalize within 24–72 h of alpelisib discontinuation, careful tapering of oral antihyperglycemic agents and insulin is required to avoid hypoglycemia [24]. Metformin should be the last antihyperglycemic therapy to be withdrawn.

## 6. Conclusions

Hyperglycemia is a common, on-target AE of alpelisib caused by α-PI3K inhibition. Even mild hyperglycemia results in hyperinsulinemia, which preclinical models suggest may reduce the efficacy of alpelisib, and more severe hyperglycemia necessitates alpelisib dose interruption and reduction, and may result in permanent discontinuation of treatment. Optimal efficacy of alpelisib can therefore only be obtained when striking a perfect balance between dosage, treatment duration, and hyperglycemia management.

Fortunately, alpelisib-induced hyperglycemia is predictable and manageable. Multidisciplinary collaboration between oncologists and endocrinologists or diabetologists, with close monitoring of blood glucose levels, and prompt antihyperglycemic treatment, even when FPG is only mildly elevated, will allow breast cancer patients to continue receiving alpelisib at the optimal dose, thereby achieving the maximum disease control possible.

## Figures and Tables

**Table 1:** Recommendations for preventive hyperglycemia management in patients scheduled to receive alpelisib.

Management Task	Recommendations
Oncologist-performed risk assessment	Assess risk of alpelisib-induced hyperglycemia based on: - FPG and HbA1c ^a^ Prediabetes: FPG 5.6 ^b^ to <7.0 mmol/L (100 ^b^ to <126 mg/dL), HbA1c 5.7 to <6.5% Diabetes: FPG ≥ 7.0 mmol/L (≥126 mg/dL), HbA1c ≥ 6.5% - History of known diabetes or gestational diabetes - BMI ≥ 30 kg/m^2^ - Age ≥ 75 years
Endocrinologist/diabetologist consultation	Refer patients with prediabetes or diabetes to an endocrinologist/diabetologist Optimize the patient’s level of blood glucose
Lifestyle advice	Advise a sugar-free, fiber-rich diet with moderate carbohydrate intake (~200 g or ~30–40% of daily calories), and principally complex carbohydrates - Assess renal function before advising a diet rich in protein Advise lifestyle changes, including exercise resulting in 55–85% of maximum heart rate, optimally for 20–30 min/day or 150 min/week, dependent on age, comorbidities, and performance status
Prophylactic medication	Metformin may be used for prevention of alpelisib-induced hyperglycemia, but there is currently no supporting evidence for this practice
Education	Educate patients and caregivers, GPs, oncologists, and endocrinologists/diabetologists on the risk and appropriate management of alpelisib-induced hyperglycemia

^a^ OGTT can be added if required. ^b^ Based on the American Diabetes Association definition of the cut-off for impaired fasting glucose [32]. BMI: body mass index; FPG: fasting plasma glucose; GP: general practitioner; HbA1c: glycated hemoglobin; OGTT: oral glucose tolerance test.

**Table 2:** Recommendations for the use of antihyperglycemic agents to treat hyperglycemia associated with alpelisib.

Treatment	Recommendations
Oral antihyperglycemic treatment	If hyperglycemia is detected (FPG > 7 mmol/L (>126 mg/dL)), an oral antihyperglycemic drug should be prescribed Evaluation of renal function, body weight, and other comorbidities before initiating antihyperglycemic treatment is important Based on oral antihyperglycemic drugs’ mechanisms of action and AEs, and on the pathophysiology of alpelisib-induced hyperglycemia, 3 groups of drugs should be preferentially considered: - Metformin up-titrated from 500 to 2000 mg/day - Pioglitazone 15–45 mg Cannot be used alone as first-line treatment because of delayed onset of effect (6–8 weeks), but can be introduced with metformin if considered necessary - SGLT2 inhibitors according to dosing recommendations Early initiation of metformin by oncologist, and referral to endocrinologist/diabetologist thereafter - Gradual metformin dose escalation from 500 to 2000 mg - In case of metformin-related diarrhea, reduce the dose or change to the XR formulation In severe cases, replace with pioglitazone or an SGLT2 inhibitor Consider discontinuation with gradual re-challenge 4–5 days later, starting with half an 850 mg tablet after dinner - Add oral antihyperglycemic treatments in a stepwise manner For grade 2 hyperglycemia ^a^, if already on metformin at a maximally tolerated dose, add pioglitazone (15–45 mg) or an SGLT2 inhibitor For grade 3 hyperglycemia ^b^, if ketones negative, up-titrate metformin from 500 to 2000 mg in combination with a second-line drug (pioglitazone or SGLT2 inhibitor), or a combination of metformin, pioglitazone, and a SGLT2 inhibitor For grade 4 hyperglycemia ^c^, if ketones negative, maximize oral antihyperglycemic therapy (metformin 2000 mg, pioglitazone 45 mg, and maximal dose SGLT2 inhibitor)
Insulin	Avoid if possible, but insulin can be used as rescue medication for 1–2 days until hyperglycemia resolves ^d^ Insulin should be initiated in cases of uncontrolled severe hyperglycemia, ketoacidosis, failure of non-insulin antihyperglycemic therapy, or concomitant acute illness In cases of grade 3/4 hyperglycemia, check ketones, and if positive, discontinue oral agents and start insulin

^a^ FPG > 8.9–13.9 mmol/L (>160–250 mg/dL); ^b^ FPG > 13.9–27.8 mmol/L (>250–500 mg/dL); ^c^ FPG ≥ 27.8 mmol/L (≥500 mg/dL); ^d^ alpelisib has a short half-life and blood glucose levels tend to normalize within 24–72 h of treatment interruption, meaning that ongoing insulin is generally not required. FPG: fasting plasma glucose; HbA1c: glycated hemoglobin; SGLT2: sodium–glucose co-transporter 2; XR: extended release.
